# Identification and characterization of the capsule depolymerase Dpo27 from phage IME-Ap7 specific to *Acinetobacter pittii*

**DOI:** 10.3389/fcimb.2024.1373052

**Published:** 2024-05-14

**Authors:** Rentao Wang, Yannan Liu, Yaqian Zhang, Shijun Yu, Hailong Zhuo, Yong Huang, Jinhui Lyu, Yu Lin, Xianglilan Zhang, Zhiqiang Mi, Youning Liu

**Affiliations:** ^1^ Senior Department of Respiratory and Critical Care Medicine, the Eighth Medical Center of Chinese PLA General Hospital, Beijing, China; ^2^ Emergency Medicine Clinical Research Center, Beijing Chao-Yang Hospital, Capital Medical University, Beijing, China; ^3^ School of Basic Medical Sciences, Anhui Medical University, Hefei, China; ^4^ State Key Laboratory of Pathogen and Biosecurity, Beijing Institute of Microbiology and Epidemiology, Beijing, China; ^5^ Department of Transfusion Medicine, The Fifth Medical Centre of Chinese PLA General Hospital, Beijing, China

**Keywords:** *Acinetobacter pittii*, capsular type, bacteriophage, capsule depolymerase, anti-virulence

## Abstract

Among the *Acinetobacter* genus, *Acinetobacter pittii* stands out as an important opportunistic infection causative agent commonly found in hospital settings, which poses a serious threat to human health. Recently, the high prevalence of carbapenem-resistant *A. pittii* isolates has created significant therapeutic challenges for clinicians. Bacteriophages and their derived enzymes are promising therapeutic alternatives or adjuncts to antibiotics effective against multidrug-resistant bacterial infections. However, studies investigating the depolymerases specific to *A. pittii* strains are scarce. In this study, we identified and characterized a capsule depolymerase, Dpo27, encoded by the bacteriophage IME-Ap7, which targets *A. pittii*. A total of 23 clinical isolates of *Acinetobacter* spp. were identified as *A. pittii* (21.91%, 23/105), and seven *A. pittii* strains with various K locus (KL) types (KL14, KL32, KL38, KL111, KL163, KL207, and KL220) were used as host bacteria for phage screening. The lytic phage IME-Ap7 was isolated using *A. pittii* 7 (KL220) as an indicator bacterium and was observed for depolymerase activity. A putative tail fiber gene encoding a polysaccharide-degrading enzyme (Dpo27) was identified and expressed. The results of the modified single-spot assay showed that both *A. pittii* 7 and 1492 were sensitive to Dpo27, which was assigned the KL220 type. After incubation with Dpo27, *A. pittii* strain was susceptible to killing by human serum; moreover, the protein displayed no hemolytic activity against erythrocytes. Furthermore, the protein exhibited sustained activity across a wide pH range (5.0–10.0) and at temperatures between 20 and 50°C. In summary, the identified capsule depolymerase Dpo27 holds promise as an alternative treatment for combating KL220-type *A. pittii* infections.

## Introduction

*Acinetobacter* spp. are a diverse group of strictly aerobic, catalase-positive, oxidase-negative, non-fermenting, Gram-negative coccobacilli (Sharma et al., 2019). This genus contains both pathogenic and non-pathogenic species that are typically found in soil, water, sewage, and food (Gomes et al., 2023). Although more than 63 officially designated species of the *Acinetobacter* genus have been identified, most are non-pathogenic organisms (Wong et al., 2017). However, certain *Acinetobacter* species, such as *Acinetobacter baumannii*, *Acinetobacter nosocomialis*, and *Acinetobacter pittii*, which are members of the *Acinetobacter calcoaceticus*–*Acinetobacter baumannii* complex (ACB complex), are pathogenic and are considered significant threats to human health (Almasaudi, 2018). Compared to *A. baumannii*, few studies have focused on *A. nosocomialis* and *A. pittii* in recent decades owing to their lower prevalence and resistance rates. However, they have garnered increasing research attention recently owing to a rise in antibiotic resistance and changes in resistance mechanisms, particularly in *A. pittii* strains (Singkham-In and Chatsuwan, 2018; Chen et al., 2019; Zhang et al., 2020; Yang et al., 2021; Ding et al., 2022; Tian et al., 2022). Additionally, a recent multicenter investigation in Japan indicated that *A. pittii* is the most significant species responsible for invasive *Acinetobacter* infections (Kiyasu et al., 2020). Reports of mortality rates associated with multidrug-resistant (MDR) *A. pittii* infections have also been on the rise in US hospitals (Fitzpatrick et al., 2015). Currently, carbapenems are the cornerstone of antimicrobial treatment for MDR *A. pittii* infections (Bassetti et al., 2021). However, resistance rates of *A. pittii* isolates to carbapenems have increased from 4.5% in 2010 to 25.8% in 2014 (Chen et al., 2019). Therefore, the development of alternative antimicrobial strategies is crucial to combat carbapenem-resistant *A. pittii* infections.

Capsular polysaccharides (CPS) on the bacterial surface play a vital role in the pathogenicity of the ACB complex by enhancing bacterial colonization, biofilm formation, and survival in mammalian tissues (Russo et al., 2010). The virulence of some bacteria decreases when CPS are removed from their surfaces (Knecht et al., 2019). Moreover, the capsules of many pathogenic bacteria reduce or inhibit complement-mediated killing (Boyce and Adler, 2000). CPS structures (CPS types) are largely determined by various polymorphisms of the chromosomal K locus (KL) in *Acinetobacter* spp. genomes (Wyres et al., 2020). To date, over 240 different gene clusters have been identified as being involved in capsule biosynthesis, each assigned a corresponding KL number (Wyres et al., 2020; Cahill et al., 2022; Kenyon and Hall, 2022). Therefore, it is imperative to identify the species and K (or KL) types of *Acinetobacter* spp.

Bacteriophages and their derived enzymes are promising therapeutic alternatives or adjuncts to antibiotics for effectively treating MDR bacterial infections (Wu et al., 2021; Blasco et al., 2022; Rao et al., 2022). Our previous studies have also indicated that phage-derived depolymerases could effectively degrade the CPS of *A. baumannii in vivo* and *in vitro*, thereby exposing non-encapsulated bacteria to immune attacks (Liu et al., 2019a, b). However, depolymerases are highly specific to host bacteria depending on the composition of the bacterial capsules (Oliveira et al., 2017). In most cases, individual strains produce only one CPS type (Timoshina et al., 2023a). While most phages encode only one or two depolymerases in a corresponding gene, some phages encode multiple depolymerases (Pan et al., 2017). Currently, depolymerases specific to K1, K2, K3, K3-v1, K9, K14, K16, K19, K26, K27, K30, K32, K37, K38, K44, K45, K47, K48, K49, K86, K87, K89, K91, K93, K116, K127, and K128 capsular types of *Acinetobacter* strains have been identified (Oliveira et al., 2017; Oliveira et al., 2019a; Domingues et al., 2021; Popova et al., 2021; Shchurova et al., 2021; Timoshina et al., 2023a, b). However, only few studies on depolymerases specific to *A. pittii* isolates have been reported to date. Thus, screening phages and exploring the activity of their depolymerases against certain capsular types of *A. pittii* may lead to effective alternative treatments. In this study, we successfully identified a capsular polysaccharide depolymerase from a lytic phage specific to KL220-type *A. pittii* and demonstrated that depolymerase Dpo27 was capable of re-sensitizing bacteria to serum. To the best of our knowledge, this is the first report of a depolymerase specific to KL220-type *A. pittii*.

## Materials and methods

### Isolate collection, species identification, and antimicrobial susceptibility testing

A total of 105 clinical isolates of *Acinetobacter* spp. were collected from five hospitals in mainland China between January 2012 and December 2018 (**Supplementary Table 1**). All strains were isolated from sputum samples of patients and cultured at 37°C in lysogeny broth (LB) media. To confirm the species level of these *Acinetobacter* isolates, a 305-bp partial *rpoB* gene was amplified, sequenced, and analyzed as described in Gundi et al. (2009). Furthermore, the antimicrobial susceptibility of *A. pittii* was determined using a Vitek 2.0 compact system (BioMérieux Clinical Diagnostics, Paris, France). Minimum inhibitory concentrations (MICs) were determined according to the breakpoints recommended by the Clinical and Laboratory Standards Institute. No humans or animals were involved in this study; therefore, ethical approval was not required.

### KL (or K) types and multilocus sequence typing of *A. pittii*

Nucleic acids of the obtained *A. pittii* strains were extracted using a High-Pure Polymerase Chain Reaction (PCR) Template Preparation Kit (Roche Diagnostics, Mannheim, Germany) according to the manufacturer’s instructions and sequenced on an Illumina HiSeq platform (Illumina, San Diego, CA, USA). The obtained raw reads were assembled *de novo* using the Unicycler v0.4.8 pipeline (Wick et al., 2017). The CPS (KL or K types) of *A. pittii* was identified using the online tool Kaptive v2.0.0, which was updated in 2021 (Wyres et al., 2020). Multilocus sequence typing (MLST) was performed via the public databases for molecular typing and microbial genome diversity (https://pubmlst.org) using seven housekeeping genes (*cpn60*, *fusA*, *gltA*, *pyrG*, *recA*, *rplB*, and *rpoB*) (Diancourt et al., 2010).

### Bacteriophage isolation and identification

Phage IME-Ap7 was isolated following the procedure described by Liu et al. (2019b). Briefly, an *A. pittii* strain with different KL types was selected as the host bacterium for screening bacteriophages. The raw sewage collected from the Fifth Medical Center of the Chinese PLA General Hospital was centrifuged to obtain the supernatant, which was then mixed with the different *A. pittii* cultures in the exponential growth phase. The mixtures were cultured at 37°C in LB medium for 6 h. After 5-min centrifugation at 10,000 rpm, the supernatants were filtered with 0.22-μm membrane filters. The phage lytic ability was determined through a double-layer agar plate assay to observe the presence of phage plaques, as previously described (Kropinski et al., 2009).

### Phage DNA extraction, sequencing, and analysis

Phage nucleic acids were extracted and purified using the High-Pure Viral RNA Kit (Roche Diagnostics). The obtained nucleic acids were sequenced and assembled, as described above. The phage genomic sequence was annotated using Rapid Annotation in Subsystem Technology (RAST; http://rast.nmpdr.org/), and the putative function of the coding sequences (CDSs) was predicted using NCBI BLASTP. The phage genome was visualized using the online software Proksee (Grant et al., 2023).

### Depolymerase cloning, expression, and purification

Based on the results of the phage genomic annotation, a gene encoding the tail fiber protein (ORF27, GenBank accession number: WRM43609.1) was predicted to have polysaccharide depolymerase activity. The sequence of ORF27 was amplified by using PCR with primers (forward: 5′-CAAATGGGTCGCGGATCCATGACAAACCCAACTTTAG-3′, reverse: 5′-GTGGTGGTGGTGCTCGAGTTATATCAACTTAACGTGA-3′) and cloned into the pET28a vector with restriction sites for *Bam*HI and *Xho*I using the *pEASY^®^*-Uni Seamless Cloning and Assembly Kit according to the manufacturer’s protocol (TransGen Biotech, Beijing, China). Clones containing inserts were selected using PCR and restriction enzyme digestion analysis and verified through DNA sequencing.

The depolymerase was expressed and purified as previously described (Liu et al., 2019b). Briefly, the recombinant plasmid containing a C-terminal hexahistidine-tag (6×His-tag) was transformed into the *Escherichia coli* BL21(DE3) and induced with 1 mM isopropyl β-D-1-thiogalactopyranoside (IPTG; Sigma-Aldrich, MO, USA) at 16°C overnight. Cells were pelleted at 10,000 rpm for 5 min and lysed by sonication (20 min with a 3-s pulse and a 4-s pause) in lysis buffer (50 mM NaH_2_PO_4_ and 300 mM NaCl, pH 8.0). Bacterial lysates were centrifuged and passed through a 0.45-μm filter. The expressed protein was purified on a gravity column with Ni-NTA resin according to the manufacturer’s instructions (Sangon Biotech, Shanghai, China). The eluted protein was collected in an 8- to 14-kDa-molecular-mass-cutoff membrane (Viskase, IL, USA) and dialyzed against a 1,000-fold volume of lysis buffer for 24 h. The molecular weight of the purified protein was determined using 10% sodium dodecyl sulfate-polyacrylamide gel electrophoresis (SDS-PAGE). The protein concentration was measured using a fluorometer (Qubit 2.0; Thermo Fisher Scientific, Waltham, MA, USA).

### Depolymerase activity

The depolymerase activity of Dpo27 against host bacteria was semi-qualitatively determined using a modified single-spot assay as previously described (Liu et al., 2019b). Briefly, *A. pittii* 7 culture in the exponential growth phase was mixed with molten soft LB agar and poured onto the surface of an LB agar plate. After solidifying, the protein dilution (0.1–2 ng) in 5 μL of PBS was dropped onto the plate, with the same volume of PBS used as a negative control. The plates were observed for the formation of translucent spots while incubating overnight at 37°C.

### Determination of the host range of phage IME-Ap7 and depolymerase Dpo27

The host range of IME-Ap7 was determined using a double-layer agar plate assay (Kropinski et al., 2009). Briefly, plates containing a 10-fold dilution of IME-Ap7 and *A. pittii* strains with different KL types were incubated overnight. The formation of singular phage plaques on bacterial lawns was used to evaluate the lytic activity and host range of isolated phage. Next, bacterial sensitivity to Dpo27 (2 ng) was determined using a modified single-spot assay, as described in the previous section.

### Extraction and purification of bacterial surface polysaccharides

Extraction and purification of bacterial exopolysaccharides (EPS) containing both CPS and liposaccharides (LPS) were conducted using a modified hot water–phenol method, as previously described (Hsieh et al., 2017). Briefly, 1 mL of *A. pittii* 7 cultured overnight in LB with 0.25% glucose was harvested and resuspended in 200 μL of double-distilled water (ddH_2_O). An equal volume of hot-water-saturated phenol (pH 6.6; Thermo Fisher Scientific) was added and vortexed vigorously. After incubating at 65°C for 20 min, the mixture was extracted and purified using chloroform to remove bacterial debris. The obtained EPS was lyophilized and stored at −20°C.

### Assessment of depolymerase activity and Alcian blue staining

The enzymatic activity of Dpo27 against bacterial surface polysaccharides was measured using the 3,5-dinitrosalicylic acid (DNS) method with minor modifications (Oliveira et al., 2017). Briefly, the EPS solution of *A. pittii* 7 (2 mg/mL) was mixed with Dpo27 or heat-inactivated Dpo27 (100°C for 15 min; 10 μg/mL) to a final volume of 1.0 mL and subsequently incubated at 37°C for 1 h; EPS or enzyme alone, respectively, served as the controls. Next, two volumes of DNS reagent (Solarbio, Beijing, China) were immediately added to each reaction mixture, and the mixtures were boiled for 5 min. The absorbance was measured at 540 nm using a Synergy HT Multi-Detection Microplate Reader (BioTek, VT, USA). The experiment was repeated at least three times.

The degradation of bacterial CPS was confirmed using Alcian blue staining, as previously described (Pan et al., 2013; Hsieh et al., 2017). Briefly, each of the mixtures described above was loaded and separated on 10% SDS-PAGE gel. After running, the gel was washed three times (5, 10, and 15 min) with the fix/wash solution (25% ethanol and 10% acetic acid in water) at room temperature and stained with 0.1% Alcian blue (Sigma-Aldrich) dissolved in the fix/wash solution for 15 min in the dark at 37°C. After the gel was destained overnight in the fixed/washed solution, CPS was visualized as a blue band.

### Stability of depolymerase in various pH values and temperatures

The effects of pH and temperature on the enzymatic activity of Dpo27 were determined as previously described (Liu et al., 2019b). The lyophilized EPS powder of *A. pittii* 7 (described in a previous section) was resuspended in 50 mM sodium acetate buffer (pH 4.0–5.0), 50 mM Na_2_HPO_4_ buffer (pH 6.0–7.0), 50 mM Tris-HCl buffer (pH 8.0–9.0), and 50 mM sodium carbonate buffer (pH 10.0–11.0) to a final concentration of 2 mg/mL, and then mixed with Dpo27 (10 μg/mL) to obtain a final volume of 1.0 mL. The mixtures were then incubated for 1 h at 37°C. To test the thermal stability of the enzyme, the EPS powder was dissolved in 50 mM Na_2_HPO_4_ buffer (pH 6.0) and then incubated with the Dpo27 (10 μg/mL) at different temperatures (20–70°C) for 1 h. EPS solution or enzyme alone was used as a control. Enzymatic activity was determined using the DNS method, as described in the previous section. All experiments were performed in triplicate.

### Human serum assay

The ability of Dpo27 to enhance bacterial susceptibility to serum killing was determined as previously described with minor modifications (Lin et al., 2017; Pan et al., 2017). Briefly, the overnight *A. pittii* 7 culture (approximately 10^7^ CFU/mL) was treated with Dpo27 (10 μg/mL) for 1 h at 37°C. The human serum from healthy volunteers or its inactivation (heated at 56°C for 30 min) was then added to the enzyme-pretreated bacteria at a volumetric ratio of 1:3, and the overnight bacteria culture was incubated with enzyme or active serum as control. After incubation for 1 h at 37°C, the mixture was serially diluted and plated for bacterial enumeration. The experiment was performed independently three times.

### Hemolysis assay

The hemolytic effect of Dpo27 on erythrocytes was evaluated as described previously, with minor modifications (Wang et al., 2015). Briefly, blood samples from healthy donors were centrifuged (1,000 rpm for 10 min) to collect serum and erythrocytes. Next, the obtained erythrocytes were washed three times and diluted to a concentration of 5% (v/v) with PBS. The erythrocytes were incubated with Dpo27 (10 μg/mL) at 37°C for 1 h with gentle shaking. The erythrocytes treated with PBS or 0.1% Triton X-100 were included as negative or positive controls, respectively. Supernatant (100 μL) was transferred to a 96-well microplate after the sample was centrifuged at 1,000 rpm for 10 min, and another 100 μL of PBS was added to the wells. The absorbance of the hemoglobin was measured at 540 nm. All experiments were repeated in triplicate.

### Statistical analysis

All experimental data are presented as means ± standard deviation (SD), and statistical analyses were performed using Prism 7 (GraphPad Software, CA, USA). One-way analysis of variance (ANOVA) was used to compare multiple groups, with *p-*values < 0.05 considered to be statistically significant.

## Results

### Species identification, antimicrobial susceptibility testing, and capsular genotyping

To determine the prevalence of different *Acinetobacter* spp. in the collected isolates, a 305-bp partial *rpoB* gene of the 105 clinical strains was sequenced and analyzed. As shown in **Supplementary Table 1**, the isolates were identified as *A. baumannii* (72.38%, 76/105), *A. pittii* (21.91%, 23/105), *A. nosocomialis* (3.81%, 4/105), and *A. soil* (1.90%, 2/105). The antimicrobial susceptibility of *A. pittii* was tested using the Vitek 2.0 compact system. The susceptibility rates of *A. pittii* to different classes of antibiotics are shown in **Supplementary Table 2**. Among the 23 A*. pittii* strains, 16 (69.57%, 16/23) were non-susceptible to one or more agents in three or more antimicrobial categories (MDR). Moreover, nine strains (39.13%, 9/23) were resistant to imipenem, a type of carbapenem. Furthermore, the KL types and MLST of 23 A*. pittii* strains were identified. As presented in **Table 1**, these isolates were assigned to KL14 (K14; 4.34%, 1/23), KL32 (K32; 4.34%, 1/23), KL38 (8.70%, 2/23), KL111 (8.70%, 2/23), KL163 (47.83%, 11/23), KL207 (17.39%, 4/23), and KL220 (8.70%, 2/23). According to the Pasteur MLST scheme, seven isolates (30.43%, 7/23) could not be typed, whereas other strains belonged to ST63 (52.17%, 12/23), ST119 (4.35%, 1/23), ST205 (8.70%, 2/23), and ST248(4.35%, 1/23).

**Table 1:** Sensitivity range of IME-Ap7/Dpo27 to various KL (or K) types and MLSTs of *A. pittii* strains.

KL (or K) type	MLST	Bacteria strain	Sensitivity to IME-Ap7	Sensitivity to Dpo27
KL14 (K14)	NT	653	–	–
KL32 (K32)	119	1316	–	–
KL38	NT	1178 1668	–	–
KL111	205	1477 1478	–	–
KL163	63	910 1475 1476 1480 1481 1482 1484 1487 1493 1494 1496	–	–
KL207	NT	1483 1488 1489 1490	–	–
KL220	248	7	+	+
	63	1492	+	+

NT, non-typable; “−”, non-sensitive; “+”, sensitive.

### Plaque observation and genome analysis of phage IME-Ap7

**Figure 1:**
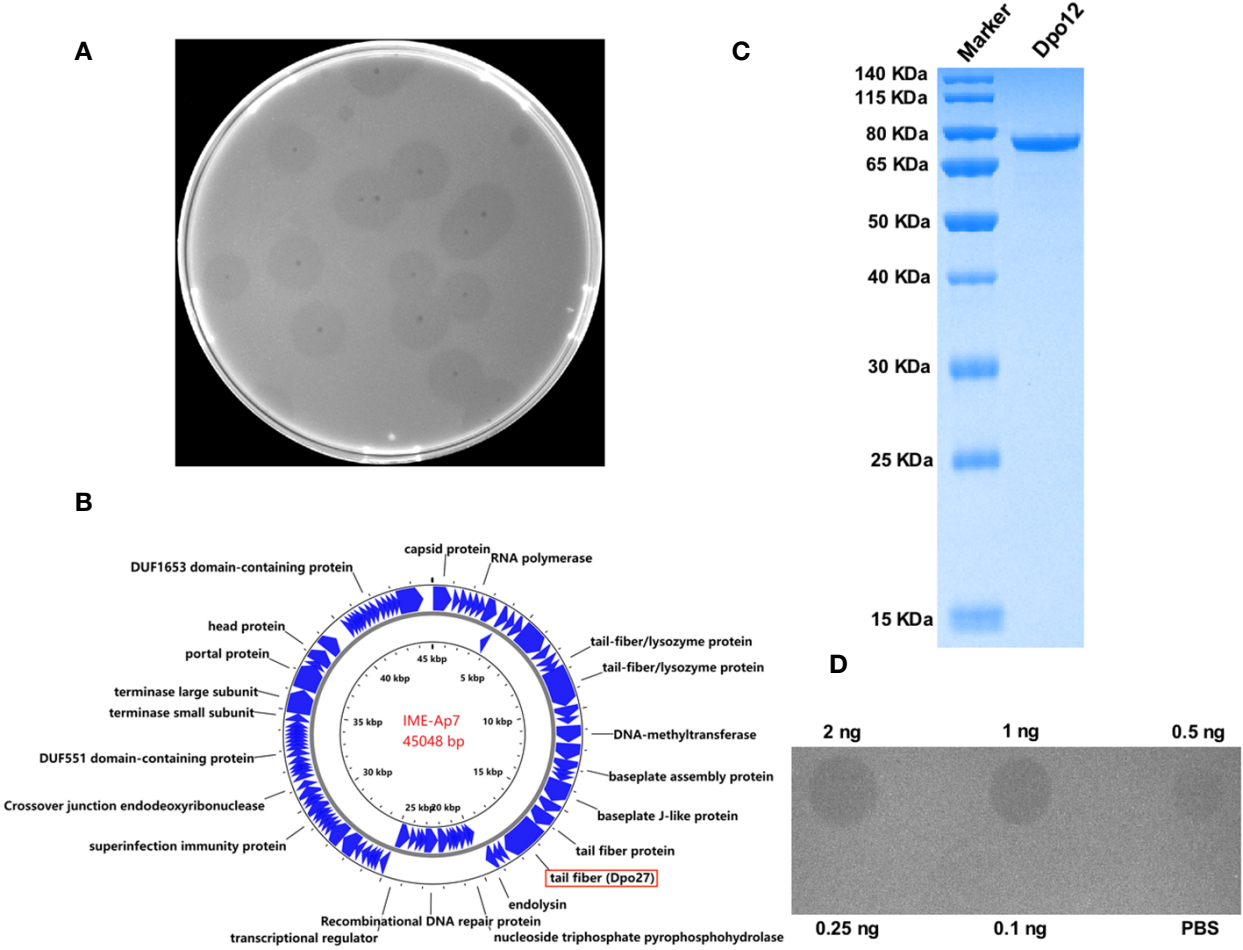
Characterization of phage IME-Ap7 and depolymerase Dpo27. **(A)** After incubation with the host bacterium *A. pittii* 7 at 37°C overnight, the phage IME-Ap7 could form clear plaques surrounded with translucent halos on the double-layer agar plate. **(B)** The annotation results from RAST and NCBI BLAST are presented using Proksee. The complete genome of phage IME-Ap7 contains 92 CDSs, 21 of which are predicted to encode a functional protein as indicated. **(C)** The purified Dpo27 migrated as a single band on 10% SDS-PAGE gel, with a molecular weight of approximately 77.60 kDa. **(D)** The polysaccharide-degrading activity of Dpo27 was determined by a modified single-spot assay, with different enzyme dilutions (0.1–2 ng) on a lawn of the host bacterium *A. pittii* 7, and PBS served as a control.

Seven *A. pittii* strains (653, 1316, 1178, 1477, 910, 1483, and 7), representative of different KL types, were used as host bacteria to screen for phages. A lytic phage, IME-Ap7, was isolated using KL220 *A. pittii* 7 as an indicator bacterium. Following incubation overnight at 37°C, the phage formed clear plaques surrounded by translucent halos on the double-layer agar plate (**Figure 1A**). Remarkably, even at room temperature, the size of translucent halos continued to increase, suggesting that some depolymerases produced from phage virions might degrade bacterial surface polysaccharides.

The phage genome was assembled after high-throughput sequencing, and the obtained sequence was deposited in GenBank under the accession number OR791279. The linear genomic sequence of phage IME-Ap7 was 45,048 bp with a G+C content of 37.9%. According to the results of RAST and NCBI BLASTP, the phage genome contains 92 CDSs, the function of 21 of which has been predicted (**Figure 1B**, **Supplementary Table 3**). ORF27 was predicted to encode a polysaccharide depolymerase with a length of 704 amino acids and a molecular weight of 77.60 kDa.

### The ORF27 displays depolymerase activity

To determine whether the predicted depolymerase exhibited activity for polysaccharide degradation, a recombinant plasmid containing the ORF27 sequence was constructed and expressed. As depicted in **Figure 1C**, the protein Dpo27 migrated as a single band on 10% SDS-PAGE gel and had an estimated size of approximately 77.60 kDa. The concentration of purified Dpo27 was determined to be 0.5 mg/mL using a fluorometer. The polysaccharide-degrading activity of Dpo27 was evaluated using a modified single-spot assay with different enzyme dilutions (0.1–2 ng). As illustrated in **Figure 1D**, the size of the semi-clear circles decreased with a reduction in Dpo27 concentration, and the halo disappeared at an enzyme concentration of 0.25 ng.

### Sensitivity range of phage IME-Ap7 and Dpo27

To determine the lytic spectrum of phage IME-Ap7, 23 A*. pittii* strains representing seven KL types were tested using a double-layer agar plate assay (**Table 1**). Phage IME-Ap7 lysed the KL220 type of *A. pittii* 7 and 1492, producing clear plaques on the bacterial lawn. The sensitivity of these isolates to Dpo27 was assessed using a modified single-spot assay. As described in **Table 1**, Dpo27 formed a translucent halo on the KL220 type of *A. pittii* 7 and 1492, indicating that the protein had the same sensitivity range as its parent phage, IME-Ap7.

### Dpo27 could degrade bacterial CPS

**Figure 2:**
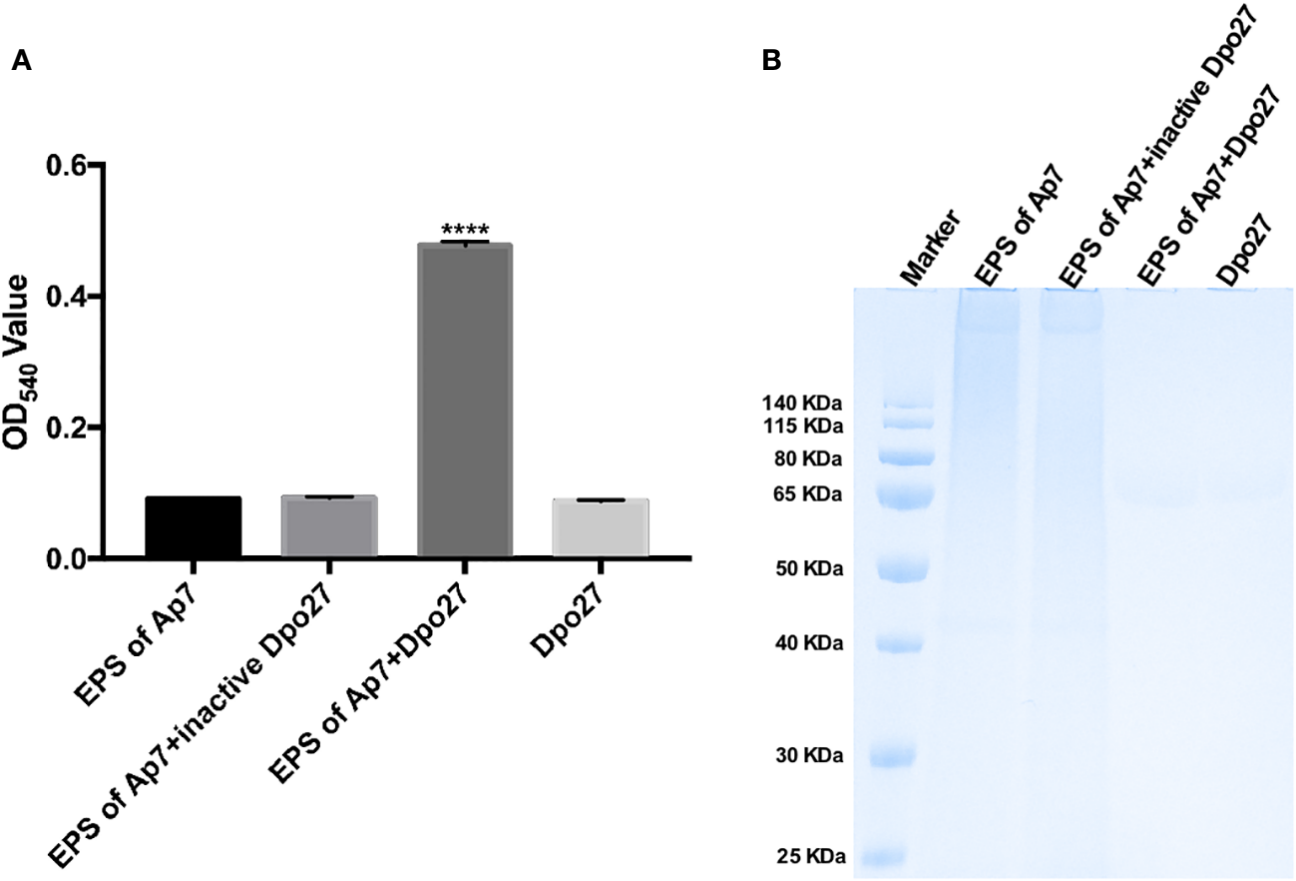
Dpo27 could effectively degrade CPS on bacterial surface. The EPS solution of *A. pittii* 7 mixed with Dpo27 or inactivated Dpo27 was incubated at 37°C for 1 h EPS or enzyme alone served as controls. **(A)** Residual EPS was quantified using the DNS method, and the absorbance of the reactions was measured at 540 nm. Data are presented as the mean ± SD (*n* = 3), and statistical analysis was performed using one-way ANOVA (****p < 0.0001). **(B)** The mixtures were examined with 10% SDS-PAGE gel and detected using Alcian blue staining.

Enzymatic activity was evaluated by monitoring the amount of reducing sugars released from the enzyme-digested bacterial surface polysaccharides. As shown in **Figure 2A**, treatment with Dpo27 resulted in the release of sugars to an OD_540_ value of 0.477 ± 0.007, which was significantly higher than that observed for treatment with heat-inactivated Dpo27 or the corresponding controls. Thus, EPS was degraded after incubation with Dpo27 (*p <* 0.0001, one-way ANOVA). The capsular polysaccharide-degrading capacity of Dpo27 was further verified using Alcian blue staining (**Figure 2B**). The results of gel electrophoresis showed that a smeared band was formed upon incubation of *A. pittii* CPS with Dpo27 when compared to the CPS alone or the CPS treated with the inactivated enzyme, indicating that the CPS of *A. pittii* 7 was degraded by Dpo27.

### Dpo27 tolerance to pH and temperature

**Figure 3:**
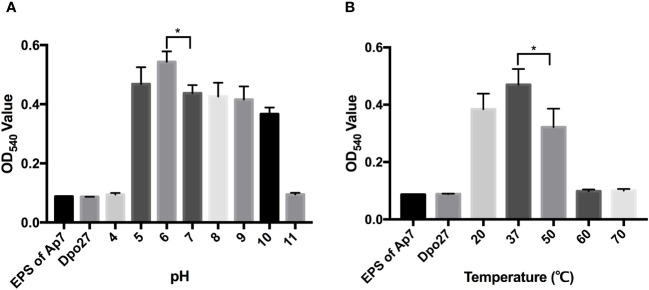
Activity of Dpo27 across a range of pH and temperatures. **(A)** The lyophilized EPS powder of *A. pittii* 7 was dissolved in 50 mM sodium acetate buffer (pH 4.0–5.0), 50 mM Na_2_HPO_4_ buffer (pH 6.0–7.0), 50 mM Tris-HCl buffer (pH 8.0–9.0), or 50 mM sodium carbonate buffer (pH 10.0–11.0) and mixed with Dpo27 for 1-h incubation at 37°C. **(B)** The EPS powder was suspended in 50 mM Na_2_HPO_4_ buffer (pH 6.0) and incubated with Dpo27 at different temperatures (20–70°C) for 1 **(h)** EPS solution or enzyme alone was used as control. The enzymatic activity was determined by the reducing sugars produced after treatment, and the absorbance of mixtures was quantified at 540 nm. All data are presented as the mean ± SD (*n* = 3), and statistical analysis was conducted using one-way ANOVA (*p < 0.05).

To determine the optimal pH for Dpo27 activity, the enzymatic activity of the protein at pH 4–11 was determined by measuring the production of reducing sugars. As shown in **Figure 3A**, Dpo27 remained active at pH 5.0–10.0. The thermal stability of Dpo27 was evaluated in 50 mM Na_2_HPO_4_ buffer (pH 6.0) using a previously described method. Dpo27 maintained enzymatic activity at temperatures ranging from 20–50°C, with an optimum temperature of 37°C (**Figure 3B**).

### Serum-sensitive assay and acute toxicity to erythrocytes of Dpo27

**Figure 4:**
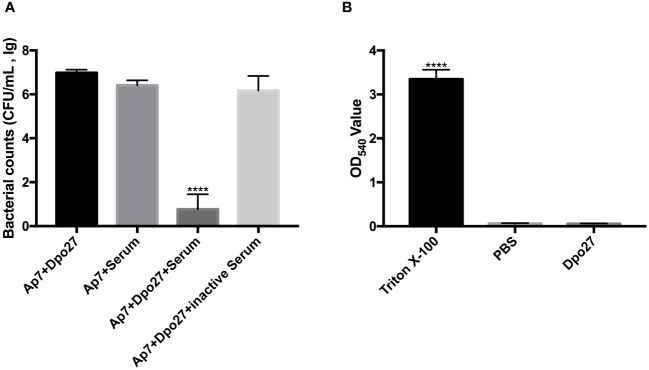
Human serum assay and hemolysis assay. **(A)** The overnight culture of *A. pittii* 7 was incubated with Dpo27 or serum, and the enzyme-pretreated bacteria were mixed with serum or inactivated serum for 1 h at 37°C. The mixtures were then serially diluted and plated for bacterial counting. **(B)** The erythrocytes were treated with Dpo27, PBS, and 0.1% Triton X-100 at 37°C for 1 h with gentle shaking at 60 rpm, respectively. Next, the absorbance of hemoglobin was measured at 540 nm. All data are presented as the mean ± SD (*n* = 3). Statistical analysis was performed using one-way ANOVA (****p < 0.0001).

To verify the capacity of Dpo27 to enhance bacterial susceptibility to killing by incubation with serum, the bacterial counts of *A. pittii* 7 were determined across different treatment groups (**Figure 4A**). *A. pittii* 7 exhibited resistance to killing by incubation with serum, as indicated by a slight reduction in viable counts of the serum-treated bacteria. However, upon treatment with depolymerase Dpo27, *A. pittii* 7 became sensitive to serum, and a significant reduction in the viable bacterial count was observed (*p <* 0.0001, one-way ANOVA). Additionally, the acute toxicity of Dpo27 toward red blood cells was assessed to further evaluate the safety of depolymerase as an antimicrobial agent. As shown in **Figure 4B**, Dpo27 displayed no hemolytic activity against erythrocytes *in vitro*.

## Discussion

Among the species of the ACB complex, *A. baumanii* stands out as a major opportunistic agent causing nosocomial infections, presenting severe manifestations such as pneumonia, urinary tract infections, bloodstream infections, and peritoneal dialysis-related peritonitis (Pailhories et al., 2018; Chopjitt et al., 2021; Yang et al., 2021; Bajaj et al., 2023). In recent years, non-*baumannii Acinetobacter* isolates have increasingly been identified in human clinical specimens and are attracting increased research attention worldwide (Chuang et al., 2011; Fitzpatrick et al., 2015). The prevalence rates of *A. pittii* are 3.2%, 5.2%, 6.4%, 9.3%, and 29% in the southern part of Thailand, South Korea, Thailand, Singapore, and Japan, respectively (Koh et al., 2012; Park et al., 2012; Chusri et al., 2014; Matsui et al., 2014; Singkham-In and Chatsuwan, 2018). In this study, a total of 23 A*. pittii* strains (21.91%, 23/105) were identified (**Supplementary Table 1**). Carbapenem-resistant *A. pittii* has recently emerged worldwide. As shown in **Supplementary Table 2**, the resistance rate of *A. pittii* to imipenem was 39.13% (9/23) in our study, which was higher than that found in Latin America (20%), Thailand (22.7%), Taiwan (33.3%), and Singapore (38.9%), but lower than that of South Korea (53.3%) (Koh et al., 2012; Park et al., 2012; Teixeira et al., 2013; Singkham-In and Chatsuwan, 2018; Chen et al., 2019). Overall, the increasing prevalence and carbapenem-resistance rate of *A. pittii* strains have begun to impose challenges in clinical therapeutics.

Several clinical trials have demonstrated the promising potential of lytic phages in treating MDR bacterial infections (Sarker et al., 2016; Jault et al., 2019; Petrovic Fabijan et al., 2020). Additionally, phage-derived proteins (such as endolysins and depolymerases) have been explored as antibacterial agents against bacterial infections *in vitro* and *in vivo* (Oliveira et al., 2017; Kim et al., 2020; Domingues et al., 2021; Pallesen et al., 2023). Previous studies have identified phages and their encoded depolymerases specific to different capsular types of *Acinetobacter* strains (Oliveira et al., 2017; Liu et al., 2019a; Oliveira et al., 2019a; Domingues et al., 2021; Popova et al., 2021; Shchurova et al., 2021; Timoshina et al., 2023a, b). However, few studies exist on depolymerases specifically targeting *A. pittii* isolates. Thus, in this study, seven *A. pittii* strains of different KL types served as indicator bacteria for phage screening. As shown in **Figure 1A**, the lytic phage IME-Ap7 was isolated using *A. pittii* 7 (KL220) as the host bacterium and was identified as possessing depolymerase activity. The phage genomes were sequenced and analyzed (**Figure 1B**, **Supplementary Table 3**). Results of NCBI BLASTN showed that the query coverage and percent identity of the phage genome sequence were 43%–62% and 87.81%–93.53%, respectively, compared to those of 41 *Acinetobacter* phages. This finding indicates that IME-Ap7 is a novel phage with a relatively lower query coverage of sequences than that of other homologous phages. Additionally, we speculated that the putative tail fiber protein (ORF27; GenBank accession number: WRM43609.1), containing a phage_tailspike_middle domain (residues 148–233 aa) at the N-terminus, may exhibit depolymerase activity. The ORF27 sequence was cloned, expressed, and purified (**Figure 1C**). As shown in **Figure 1D**, Dpo27 was active against the host strain, *A. pittii* 7, at a minimum concentration of 0.5 ng. This finding aligns with our previous report, where ORF71 of phage IME-AB2 (GenBank accession number: YP_009592222.1), containing a phage_tailspike_middle domain (residues 161–238 aa) in the N-terminus, was identified as a depolymerase (Chen et al., 2022).

Although bacteriophages or their derived proteins can effectively and safely control bacterial infections as potential therapeutic agents, their wide application in clinical settings is limited due to their narrow spectrum and high specificity (Oliveira et al., 2017). Finding a bacteriophage or its derivatives with a wide host range or creating a cocktail of bacteriophage-derived antibacterial agents targeting different types of bacteria may be a feasible strategy in the future. In the present study, both phage IME-Ap7 and depolymerase Dpo27 were shown to target the KL220-type *A. pittii* strains (7 and 1492), indicating an extremely narrow host range (**Table 1**). This trait has also been observed in capsular depolymerases from phages infecting *Klebsiella pneumoniae* and *E. coli*, which are often restricted to one or two K types (Lin et al., 2014; Majkowska-Skrobek et al., 2016; Lin et al., 2017; Liu et al., 2020). *A. pittii* 7 and 1492 were resistant to imipenem and belonged to strains ST248 and ST63, respectively. Both MLSTs have often been identified in other carbapenem-resistant *A. pittii* isolates (Chopjitt et al., 2021; Yang et al., 2021; Tian et al., 2023), underscoring the need to focus on the prevalence of this type of *A. pittii*. Although the sensitivity ranges of IME-Ap7 and Dpo27 were determined only using the 105 clinical isolates of *Acinetobacter* spp. collected in this study (data not shown), we hypothesize that this phage or depolymerase is specific to most *Acinetobacter* strains of the KL220 type, according to the results of Oliveira et al. (2017). Furthermore, considering the host specificity of phage and depolymerase, the phage IME-Ap7 and depolymerase Dpo27 could be used to rapidly identify the capsular type of *Acinetobacter* spp (Timoshina et al., 2023a).

To determine the hydrolytic activity of Dpo27 under different conditions, the EPS extracted from the bacterial surface was degraded and quantified using the DNS method. As described in **Figure 3**, the Dpo27 was active at various pH values (5.0–10.0) and temperatures (20–50°C). Similarly, the B9gp69, Dpo48, and K2 maintained activity under moderately acidic or alkaline conditions (pH 5.0–9.0) (Oliveira et al., 2019a; Liu et al., 2019b; Oliveira et al., 2019b). However, DpoMK34 has a broader pH range (4–11) compared to that of the other four depolymerases (Abdelkader et al., 2022). Furthermore, DpoMK34 had a similar temperature range (20–50°C) to Dpo27, which was narrower than that of B9gp69 (20–80°C), Dpo48 (20–70°C), and K2 (20–70°C) (Oliveira et al., 2019a; Liu et al., 2019b; Oliveira et al., 2019b). In summary, Dpo27 ensured high efficacy over a relatively broad range of pH values and moderate temperatures.

CPS is an important virulence factor that helps bacteria evade host immunity; therefore, the degradation of CPS deprives encapsulated bacteria of a vital shield, making them more susceptible to the host immune-defense system (Liu et al., 2019a, b, 2020). In this study, the survival counts of bacteria showed that the group of enzyme-pretreated bacteria plus active serum decreased by ~6 log compared to that of the untreated bacteria plus serum (**Figure 4A**). This result indicated that Dpo27 could enhance bacterial susceptibility to killing by human serum, which was also previously verified (Liu et al., 2019b; Abdelkader et al., 2022). In addition, the enzyme mixed with serum could not completely eradicate all bacteria, and similar observations have also been reported in previous studies (Liu et al., 2019b, 2020; Abdelkader et al., 2022). According to our previous research, incomplete bacterial eradication might be attributed to the presence of a subpopulation of bacteria that are susceptible to depolymerases but resist serum complement-mediated killing (Liu et al., 2019b, 2020). Notably, Dpo27 displayed no hemolytic activity against erythrocytes (**Figure 4B**), which suggests that the enzyme has the potential as a therapeutic agent and warrants further exploration of its antibacterial properties *in vivo*.

In conclusion, the capsule depolymerase, Dpo27, from phage IME-Ap7 is specific to KL220-type *A. pittii* strains. The enzyme could effectively strip the CPS on bacterial surfaces and maintained activity across a wide range of pH values (5.0–10.0) and temperatures (20–50°C). Moreover, the enzyme enhanced the sensitivity of bacteria to human serum, but had no hemolytic effect on erythrocytes. These results suggest that Dpo27 has the potential to be developed as an alternative treatment for the prevention and control of MDR KL220-type *A. pittii* strains.

## Data availability statement

The datasets presented in this study can be found in online repositories. The names of the repository/repositories and accession number(s) can be found below: https://www.ncbi.nlm.nih.gov/genbank/, OR791279.

## Author contributions

RW: Writing – review & editing, Conceptualization. YaL: Conceptualization, Writing – original draft. YZ: Writing – review & editing, Methodology. SY: Writing – review & editing, Methodology. HZ: Writing – review & editing, Methodology. YH: Writing – review & editing, Software. JL: Writing – review & editing, Methodology. YuL: Writing – review & editing, Software. XZ: Writing – review & editing, Software. ZM: Writing – original draft, Resources, Conceptualization. YoL: Writing – review & editing, Resources, Conceptualization.
