# Supplementary material for: Identification and characterization of the capsule depolymerase Dpo27 from phage IME-Ap7 specific to Acinetobacter pittii
Source: Front Cell Infect Microbiol. 2024 May 14;14:1373052. doi: 10.3389/fcimb.2024.1373052 (PMC11130378; doi:10.3389/fcimb.2024.1373052)
Supplement: Supplementary file 1 [file Table_1.docx]

| ***Acinetobacter* spp.** | **Strain** | **Collection** |
| --- | --- | --- |
| *A. baumannii* (72.38%, 76/105) | 178, 187, 220, 225, 237, 274, 291, 570, 587, 593, 595, 597, 623, 629, 631, 654, 658, 662, 678, 698, 704, 913, 920, 1026, 1027, 1190, 1194, 1208, 1212, 1292 | The Fifth Medical Center, Chinese PLA General Hospital, Beijng, China |
|  | 1033, 1041, 1044, 1058, 1061, 1076, 1084, 1092, 1227, 1230, 1234, 1250, 1258, 1259, 1277, 1280, 1583 | 967th Hospital of the Joint Logistics Support Force of PLA, Dalian, China. |
|  | 1485, 1486, 1495 | The First Medical Center, Chinese PLA General Hospital, Beijng, China |
|  | 1667, 1669, 167, 1678, 1682, 1683, 1684, 1685, 1686, 1687, 1688, 1689, 1690 | Strategic Support Force Medical Center, Beijing, China. |
|  | 2878, 2879, 2880, 2881, 2882, 2883, 2884, 2885, 2886, 2887, 2888, 2889, 2890 | The Affiliated Hospital of Qingdao University, Qingdao, China. |
| *A. pittii*  (21.91%, 23/105) | 653, 910, 1178, 1316 | The Fifth Medical Center, Chinese PLA General Hospital, Beijng, China |
|  | 7, 1475, 1476, 1477, 1478, 1480, 1481, 1482, 1483, 1484, 1487, 1488, 1489, 1490, 1492, 1493, 1494, 1496 | The First Medical Center, Chinese PLA General Hospital, Beijng, China |
|  | 1668 | Strategic Support Force Medical Center, Beijing, China. |
| *A. nosocomialis* (3.81%, 4/105) | 295 | The Fifth Medical Center, Chinese PLA General Hospital, Beijng, China |
|  | 1497, 1498, 1500 | The First Medical Center, Chinese PLA General Hospital, Beijng, China |
| *A. soil*  (1.90%, 2/105) | 1491, 1499 | The First Medical Center, Chinese PLA  General Hospital, Beijng, China |

**Supplementary Table 1. The species identification of *Acinetobacter* spp. isolates**
