# Supplementary material for: Identification and characterization of the capsule depolymerase Dpo27 from phage IME-Ap7 specific to Acinetobacter pittii
Source: Front Cell Infect Microbiol. 2024 May 14;14:1373052. doi: 10.3389/fcimb.2024.1373052 (PMC11130378; doi:10.3389/fcimb.2024.1373052)
Supplement: Supplementary file 2 [file Table_2.doc]

**Supplementary Table 2. Susceptibility rates of *A. pittii* to different categories of antibiotics**

| **Antimicrobial category** | **Antimicrobial agent** | **Susceptible (%)** | **Intermediate (%)** | **Resistant (%)** |
| --- | --- | --- | --- | --- |
| Penicillins + *β*-lactamase inhibitors | Ampicillin-sulbactam | 78.26 (18/23) | 8.70 (2/23) | 13.04 (3/23) |
| Extended-spectrum cephalosporins | Cefepime | 47.83 (11/23) | 13.04 (3/23) | 39.13 (9/23) |
|  | Ceftazidime | 34.78 (8/23) | 56.52 (13/23) | 8.70 (2/23) |
|  | Ceftriaxone | 4.35 (1/23) | 65.22 (15/23) | 30.43 (7/23) |
| Antipseudomonal carbapenems | Imipenem | 43.48 (10/23) | 17.39 (4/23) | 39.13 (9/23) |
| Aminoglycosides | Gentamicin | 52.18 (12/23) | 13.04 (3/23) | 34.78 (8/23) |
|  | Tobramycin | 78.26 (18/23) | 17.39 (4/23) | 4.35 (1/23) |
| Folate pathway inhibitors | Trimethoprim-sulphamethoxazole | 39.13 (9/23) | 0 (0/23) | 60.87 (14/23) |
| Antipseudomonal fluoroquinolones | Ciprofloxacin | 60.87 (14/23) | 0 (0/23) | 39.13 (9/23) |
|  | Levofloxacin | 69.57 (16/23) | 4.35 (1/23) | 26.08 (6/23) |
