# Supplementary material for: Identification and characterization of the capsule depolymerase Dpo27 from phage IME-Ap7 specific to Acinetobacter pittii
Source: Front Cell Infect Microbiol. 2024 May 14;14:1373052. doi: 10.3389/fcimb.2024.1373052 (PMC11130378; doi:10.3389/fcimb.2024.1373052)
Supplement: Supplementary file 3 [file Table_3.docx]

**Supplementary Table 3. The list of ORFs in the genome of phage IME-Ap7 and their putative functions**

| **ORF** | **Location (nt)^a^** | **Product**  **Length (aa)^b^** | **Homologue** | **Accession number** | **Sequence**  **query cover (%)^c^** | **Sequence**  **identity (%)^c^** |
| --- | --- | --- | --- | --- | --- | --- |
| 1 | 26..1018 | 330 | putative capsid protein of Acinetobacter phage AB1 | WRM43583.1 | 100 | 100 |
| 2 | 1098..1448 | 116 | hypothetical protein of Acinetobacter phage vB_AbM_WUPSU | WRM43584.1 | 100 | 96.55 |
| 3 | 1451..1900 | 149 | hypothetical protein of Acinetobacter phage BUCT628 | WRM43585.1 | 100 | 88.59 |
| 4 | 1973..2101 | 42 | hypothetical protein of Acinetobacter phage vB_AbaM_IME512 | WRM43586.1 | 100 | 95.24 |
| 5 | 2134..2571 | 145 | putative RNA polymerase of Acinetobacter phage P711 | WRM43587.1 | 100 | 96.55 |
| 6 | 2568..2753 | 61 | hypothetical protein of Acinetobacter phage vB_AbM_WUPSU | WRM43588.1 | 100 | 98.36 |
| 7 | 2815..3522 | 235 | hypothetical protein of Acinetobacter phage BS46 | WRM43589.1 | 99 | 94.02 |
| 8 | 3525..3647 | 40 | hypothetical protein of Acinetobacter phage Brutus | WRM43590.1 | 80 | 69 |
| 9 | 3677..4180 | 167 | hypothetical protein of Acinetobacter phage Bphi-R2919 | WRM43591.1 | 100 | 97.01 |
| 10 | 4177..4590 | 137 | hypothetical protein of Acinetobacter phage Abp95 | WRM43592.1 | 100 | 96.35 |
| 11 | 4587..5084 | 165 | hypothetical protein of Acinetobacter phage vB_AbaM_IME284 | WRM43593.1 | 100 | 93.33 |
| 12 | 5074..6537 | 487 | hypothetical protein of Acinetobacter phage vB_AbaM_IME512 | WRM43594.1 | 100 | 98.97 |
| 13 | 6550..6999 | 149 | hypothetical protein of Acinetobacter phage vB_AbaM_IME284 | WRM43595.1 | 100 | 99.33 |
| 14 | 7045..7470 | 141 | hypothetical protein of Acinetobacter phage BUCT628 | WRM43596.1 | 100 | 99.29 |
| 15 | 7500..7712 | 70 | putative tail-fiber/lysozyme protein of Acinetobacter phage YMC-13-01-C62 | WRM43597.1 | 100 | 100 |
| 16 | 7715..9763 | 682 | putative tail-fiber/lysozyme protein of Acinetobacter phage HZY2308 | WRM43598.1 | 100 | 92.67 |
| 17 | 9771..10388 | 205 | hypothetical protein of Acinetobacter phage LZ35 | WRM43599.1 | 100 | 96.59 |
| 18 | 10385..10660 | 91 | hypothetical protein of Acinetobacter phage Arbor | WRM43600.1 | 100 | 94.51 |
| 19 | 10791..11684 | 297 | DNA (cytosine-5-)-methyltransferase of Thermovibrio guaymasensis | WRM43601.1 | 100 | 99.66 |
| 20 | 11720..12610 | 296 | hypothetical protein of [Acinetobacter phage vB_AbaM_BP10](https://www.ncbi.nlm.nih.gov/Taxonomy/Browser/wwwtax.cgi?id=2985293) | WRM43602.1 | 100 | 97.30 |
| 21 | 12591..13238 | 215 | putative baseplate assembly protein of Acinetobacter phage BUCT628 | WRM43603.1 | 100 | 97.21 |
| 22 | 13241..13387 | 48 | hypothetical protein of Acinetobacter phage Scipio | WRM43604.1 | 100 | 97.92 |
| 23 | 13384..13737 | 117 | hypothetical protein of Acinetobacter phage Abp95 | WRM43605.1 | 100 | 99.15 |
| 24 | 13734..14918 | 394 | putative baseplate J-like protein of Acinetobacter phage vB_AbaM_BP10 | WRM43606.1 | 100 | 99.24 |
| 25 | 14918..15544 | 208 | hypothetical protein of Acinetobacter phage Abp95 | WRM43607.1 | 100 | 97.12 |
| 26 | 15537..16367 | 276 | putative tail fiber protein of Acinetobacter phage AB1 | WRM43608.1 | 100 | 77.06 |
| 27 | 16369..18483 | 704 | putative tail fiber of Acinetobacter phage vB_AbaM-IME-AB2 (Dpo27) | WRM43609.1 | 35 | 61.45 |
| 28 | 18577..18897 | 106 | hypothetical protein of Acinetobacter phage Arbor | WRM43610.1 | 100 | 98.11 |
| 29 | 18881..19153 | 90 | hypothetical protein of Acinetobacter phage Brutus | WRM43611.1 | 98 | 100 |
| 30 | 19143..19655 | 170 | putative endolysin of Acinetobacter phage vB_AbaM-IME-AB2 | WRM43612.1 | 100 | 97.06 |
| 31 | 19754..19975 | 73 | hypothetical protein of Thermovibrio guaymasensis | WRM43613.1 | 100 | 98.63 |
| 32 | 19972..20202 | 76 | hypothetical protein of Acinetobacter phage BUCT628 | WRM43614.1 | 100 | 92.11 |
| 33 | 20195..20740 | 181 | putative nucleoside triphosphate pyrophosphohydrolase of Acinetobacter phage Cato | WRM43615.1 | 100 | 99.45 |
| 34 | 20740..20904 | 54 | hypothetical protein of Acinetobacter phage Cato | WRM43616.1 | 100 | 100 |
| 35 | 20901..21104 | 67 | hypothetical protein of Acinetobacter phage vB_ApiM_IME-Ap7 | WRM43617.1 | 100 | 100 |
| 36 | 21101..21394 | 97 | hypothetical protein of Acinetobacter phage vB_AbaM_AB3P2 | WRM43618.1 | 100 | 98.97 |
| 37 | 21395..22150 | 251 | hypothetical protein of Acinetobacter phage vB_AbaM_IME285 | WRM43619.1 | 100 | 97.21 |
| 38 | 22147..23046 | 299 | recombinational DNA repair protein of Acinetobacter phage vB_AbaM_IME284 | WRM43620.1 | 100 | 98.33 |
| 39 | 23043..23225 | 60 | hypothetical protein of Acinetobacter phage AP22 | WRM43621.1 | 100 | 98.33 |
| 40 | 23225..23557 | 110 | hypothetical protein of Acinetobacter phage vB_AbaM_AB3P2 | WRM43622.1 | 100 | 98.18 |
| 41 | 23652..23999 | 115 | hypothetical protein of Acinetobacter phage Cato | WRM43623.1 | 70 | 91.36 |
| 42 | 24066..24878 | 270 | putative transcriptional regulator of Acinetobacter phage vB_AbaM_fThrA | WRM43624.1 | 100 | 73.48 |
| 43 | 24980..25405 | 141 | hypothetical protein of Acinetobacter phage BUCT628 | WRM43625.1 | 100 | 100 |
| 44 | 25508..25696 | 62 | hypothetical protein of Acinetobacter phage vB_ApiM_IME-Ap7 | WRM43626.1 | 100 | 100 |
| 45 | 25693..25854 | 53 | hypothetical protein of Acinetobacter phage vB_AbaM_BP10 | WRM43627.1 | 100 | 100 |
| 46 | 25851..26093 | 80 | hypothetical protein of Acinetobacter phage vB_AbaM_IME284 | WRM43628.1 | 100 | 95 |
| 47 | 26090..26446 | 118 | hypothetical protein of Acinetobacter phage vB_AbaM_IME285 | WRM43629.1 | 100 | 97.46 |
| 48 | 26533..26778 | 81 | hypothetical protein of Acinetobacter phage phiAC-1 | WRM43630.1 | 83 | 79.41 |
| 49 | 26786..27649 | 287 | hypothetical protein of Acinetobacter phage vB_ApiM_IME-Ap7 | WRM43631.1 | 100 | 100 |
| 50 | 27649..28491 | 280 | hypothetical protein of Acinetobacter phage vB_ApiM_IME-Ap7 | WRM43632.1 | 100 | 100 |
| 51 | 28478..28681 | 67 | hypothetical protein of Thermovibrio guaymasensis | WRM43633.1 | 100 | 95.36 |
| 52 | 28678..28986 | 102 | hypothetical protein of Acinetobacter phage vB_AbaM_AB3P2 | WRM43634.1 | 100 | 94.12 |
| 53 | 28983..29156 | 57 | hypothetical protein of Acinetobacter phage BUCT628 | WRM43635.1 | 100 | 98.25 |
| 54 | 29227..29439 | 70 | immunity to superinfection of Acinetobacter phage vB_AbaM-IME-AB2 | WRM43636.1 | 100 | 100 |
| 55 | 29436..29549 | 37 | hypothetical protein of Acinetobacter phage Cato | WRM43637.1 | 100 | 91.89 |
| 56 | 29546..30130 | 194 | hypothetical protein of Acinetobacter phage BUCT628 | WRM43638.1 | 98 | 96.88 |
| 57 | 30127..30291 | 54 | hypothetical protein of Acinetobacter phage LZ35 | WRM43639.1 | 100 | 98.15 |
| 58 | 30288..30473 | 61 | hypothetical protein of Acinetobacter phage vB_ApiM_IME-Ap7 | WRM43640.1 | 100 | 100 |
| 59 | 30470..31000 | 176 | hypothetical protein of Acinetobacter phage vB_AbaM_AB3P2 | WRM43641.1 | 96 | 97.04 |
| 60 | 30984..31346 | 120 | crossover junction endodeoxyribonuclease of Acinetobacter phage AP22 | WRM43642.1 | 100 | 95.83 |
| 61 | 31483..31722 | 79 | hypothetical protein of Acinetobacter phage BUCT628 | WRM43643.1 | 100 | 97.47 |
| 62 | 31786..32004 | 72 | hypothetical protein of Acinetobacter phage vB_AbaM_AB3P2 | WRM43644.1 | 100 | 40.54 |
| 63 | 32010..32132 | 40 | hypothetical protein of Acinetobacter phage vB_AbaM_IME512 | WRM43645.1 | 100 | 95 |
| 64 | 32125..32343 | 72 | hypothetical protein of Acinetobacter phage AP22 | WRM43646.1 | 100 | 95.83 |
| 65 | 32340..32666 | 108 | hypothetical protein of Acinetobacter phage vB_AbaM_BP10 | WRM43647.1 | 100 | 93.52 |
| 66 | 32666..33076 | 136 | DUF551 domain-containing protein of Acinetobacter baumannii | WRM43648.1 | 100 | 68.49 |
| 67 | 33073..33282 | 69 | hypothetical protein of Acinetobacter baumannii | WRM43649.1 | 100 | 98.55 |
| 68 | 33279..33491 | 70 | hypothetical protein of Acinetobacter | WRM43650.1 | 100 | 100 |
| 69 | 33488..33679 | 63 | hypothetical protein of Acinetobacter calcoaceticus/baumannii complex | WRM43651.1 | 100 | 100 |
| 70 | 33681..33881 | 66 | hypothetical protein of Acinetobacter phage vB_AbaM_IME284 | WRM43652.1 | 100 | 87.88 |
| 71 | 33878..34015 | 45 | hypothetical protein of Acinetobacter phage AP22 | WRM43653.1 | 100 | 95.56 |
| 72 | 34012..34254 | 80 | hypothetical protein of Acinetobacter phage vB_AbaM_fThrA | WRM43654.1 | 100 | 97.50 |
| 73 | 34244..34408 | 54 | hypothetical protein of Acinetobacter phage vB_AbaM_BP10 | WRM43655.1 | 100 | 92.59 |
| 74 | 34475..34906 | 143 | putative terminase small subunit of Acinetobacter phage AP22 | WRM43656.1 | 100 | 98.60 |
| 75 | 34887..36188 | 433 | putative terminase large subunit of Acinetobacter phage vB_AbaM_fThrA | WRM43657.1 | 100 | 99.08 |
| 76 | 36191..37618 | 475 | putative portal protein of Acinetobacter phage Brutus | WRM43658.1 | 99 | 97.89 |
| 77 | 37605..37775 | 56 | hypothetical protein of Acinetobacter phage vB_AbM_WUPSU | WRM43659.1 | 100 | 100 |
| 78 | 37776..38546 | 256 | putative head protein of Acinetobacter phage Ab59 | WRM43660.1 | 100 | 98.44 |
| 79 | 38613..39419 | 268 | hypothetical protein of Acinetobacter phage Ab31 | WRM43661.1 | 100 | 96.64 |
| 80 | 40194..40523 | 109 | hypothetical protein of Acinetobacter phage Cato | WRM43662.1 | 99 | 86.11 |
| 81 | 40520..40726 | 68 | hypothetical protein of Acinetobacter phage Brutus | WRM43663.1 | 100 | 98.53 |
| 82 | 40728..40919 | 63 | hypothetical protein of Acinetobacter phage Abp95 | WRM43664.1 | 100 | 98.41 |
| 82 | 40916..41083 | 55 | hypothetical protein of Acinetobacter phage Abp95 | WRM43665.1 | 100 | 90.91 |
| 84 | 41084..41269 | 61 | hypothetical protein of Acinetobacter phage vB_AbaM_AB3P2 | WRM43666.1 | 100 | 90.16 |
| 85 | 41266..41679 | 137 | hypothetical protein of Acinetobacter pittii | WRM43667.1 | 100 | 99.27 |
| 86 | 41669..41851 | 60 | DUF1653 domain-containing protein of Acinetobacter baumannii | WRM43668.1 | 100 | 100 |
| 87 | 41844..42161 | 105 | hypothetical protein of Acinetobacter pittii | WRM43669.1 | 100 | 99.05 |
| 88 | 42163..42555 | 130 | hypothetical protein of Acinetobacter baumannii | WRM43670.1 | 79 | 57.26 |
| 89 | 42552..42716 | 54 | hypothetical protein of Acinetobacter phage WCHABP12 | WRM43671.1 | 100 | 77.78 |
| 90 | 42794..43000 | 68 | hypothetical protein of Acinetobacter phage YMC-13-01-C62 | WRM43672.1 | 100 | 100 |
| 91 | 43051..43230 | 59 | hypothetical protein of Acinetobacter phage vB_AbaM_fThrA | WRM43673.1 | 91 | 75.93 |
| 92 | 43217..44569 | 450 | hypothetical protein of Acinetobacter phage vB_AbaM_IME284 | WRM43674.1 | 99 | 95.75 |

^a^ nt, nucleotide; ^b^ aa, amino acid; ^c^ determined by BLAST-P.
